# Does diabetes mellitus mitigate the gender gap in COVID-19 mortality?

**DOI:** 10.1530/EJE-21-0721

**Published:** 2021-09-10

**Authors:** Alexandra Kautzky-Willer

**Affiliations:** 1 Department of Internal Medicine III, Clinical Division of Endocrinology and Metabolism, Gender Medicine Unit, Medical University of Vienna, Vienna, Austria

## Abstract

In this SARS-COV2-pandemic, diabetes mellitus (DM) soon emerged as one of the most prominent risk factors for a severe course of corona virus disease-2019 (COVID-19) and increased mortality due to hyperglycemia/insulin resistance, obesity, inflammation, altered immune status, and cardiovascular complications. In general, men are at a higher risk of severe or fatal COVID-19 disease irrespective of age, region and despite comparable infection rates in both sexes. In COVID-19, there is also a male predominance among hospitalized patients with diabetes, however, overall, data among patients with diabetes are ambiguous so far. Of note, similar to cardiovascular complications, women with type 2 diabetes (DM2) appear to lose their biological female advantage resulting in comparable death rates to those of men. The complex interplay of biological and behavioral factors, which may put men at greater risk of a severe or fatal course of COVID-19, and gender-related psychosocial factors, which may cause disadvantage to women concerning the infection rates, might explain why sex-disaggregated data among infected patients with diabetes are conflicting. Better knowledge on biological factors leading to functionally different immune responses and of gender-sensitive sociocultural determinants of COVID-19 infection rates may help to optimize prevention and management in the high-risk groups of men and women with diabetes.

Soon after the outbreak of the corona virus disease-2019 (COVID-19), diabetes emerged as one of the most prominent and prevalent risk factors for a severe course and higher mortality in this pandemic. Hyperglycemia/insulin resistance, obesity, hypertension, and the metabolic syndrome, which are all common features of patients with type 2 diabetes (DM2) and related to metainflammation, increased the risk of intensive-care-unit (ICU) admission, invasive mechanical ventilation (IMV), and mortality. However, it soon became apparent that type 1 diabetes (DM1), which is associated with an altered immune status and hyperglycemia, is also related to increased mortality. A nationwide analysis in England revealed that patients with DM2 had a 1.8-fold increased risk and those with DM1 a 2.9-fold higher risk of in-hospital death compared to non-diabetic subjects, even after adjustment for age, sex, socioeconomic deprivation, ethnicity, region, and previous cardiovascular hospital treatments (1).

Odds for in-hospital deaths for people with COVID-19 were almost two-fold higher in men than in women. There were differences in the risk of in-hospital mortality with COVID-19 depending on diabetes type, age, sex, and ethnicity, revealing a relatively higher effect of having DM2 on COVID-19-related mortality for women than men. However, many studies were not able to differentiate between the different types of diabetes mellitus (DM), thus, mostly referring to DM2, which represents the majority of hyperglycemic patients. Similar outcomes were recently reported by a cohort study of the whole population of Scotland, however, after adjustment for age and diabetes duration, no difference in risk for fatal or ICU-treated COVID-19 was found between diabetes types (2).

Diabetes was associated with a 40% higher risk for ICU admission or mortality which was similar in both men and women. In patients with DM, irrespective of diabetes type, male sex significantly increased the risk of fatal or ICU-treated COVID-19. Similarly, smoking, living in residential care or a deprived area, presence of microvascular complications, worse glycemic control, a history of diabetic ketoacidosis or hypoglycemia hospitalization showed to be a negative predictor in COVID-19 prognosis amongst patients with DM (3). A multicentre study from Austria, including hospitalized COVID-19 patients with prediabetes, DM1 or DM2, found that neither sex nor BMI were risk factors for mortality, suggesting a risk score model including only age, C-reactive protein (CRP), kidney and liver function, and inflammation parameters for hyperglycemic patients (4). Interestingly, mortality rates in prediabetes did not differ from overt diabetes.

Overall, up to now, sex-disaggregated data are scarcely reported within the topic of DM. The CORONADO initiative is a nationwide observational multicentre cohort study of DM hospitalized for COVID-19 in France. The composite primary outcome was tracheal intubation for IMV and/or death within 7 days of admission. Secondary outcomes included death, intubation, admission to ICUs and hospital discharge, with a 28-days follow-up (5). The demographic data of the cohort were comparable to international reports (64% men, mean age: 70 years and BMI: 28 kg/m^2^). The investigators aimed to phenotype the patients’ characteristics and then identify the prognostic factors. The mortality rate was 11% at 7 days and 21% at follow-up, which corresponds to other reports. Younger age, metformin therapy, and longer symptom duration were related to a greater probability of discharge, whereas history of microvascular complications, anticoagulant therapy, dyspnoea at admission, inflammatory parameters (white cell count, CRP), and aspartate aminotransferase were associated with a lower chance of discharge, thus, representing a higher mortality risk. Of note, the sex ratio was neither related to discharge nor to death. Furthermore, female sex was associated with a lower mortality risk and a higher probability to be discharged.

Another sex-specific analysis of hospitalized COVID-19 patients in New Orleans (61% women, median age: 60 years) revealed that rates of ICU, IMV, and in-hospital deaths were similar in men and women, which is in a stark contrast to most reports from other regions (6).

The authors also highlighted sex disparities in clinical determinants of severe outcomes. Obesity, diabetes, and hypertension were more prevalent in women; diabetes, chronic kidney disease, increased neutrophile-to-lymphocyte ratio, and ferritin independently predicted death in women only. In women, increased innate immune proinflammatory cytokines were associated with an unfavourable disease progression, which suggests a relatively greater impact of exaggerated innate immune response on the development of severe disease in women than men. In contrast, elevated D-dimer, a marker of hypercoagulability, independently predicted severe disease or death in men only. The latest sex-disaggregated* post hoc* analysis of the CORONADO study published in this journal reported sex disparities in severe COVID-19 outcomes of hospitalized patients with DM. After multiple adjustments, female sex was inversely related to the primary outcome (IMV and/or death), mortality or ICU admission at day 7, and ICU admission at day 28 (7). In addition, chronic obstructive pulmonary disease (COPD) predicted death in women only. This is in line with data from New Orleans, where COPD was associated with ICU and IMV in women only (6). Additionally, older age, a higher prevalence of obesity, and gastrointestinal symptoms were consistently seen among women compared to men in both studies. In CORONADO, lymphocytopenia independently predicted mortality only in women; thrombocytopenia and hyperglycaemia predicted mortality only in men. Overall, early incidence of severe outcomes was lower in women but total in-hospital mortality was similar between the sexes, suggesting that DM could largely offset the female protection from severe COVID-19 (6).

In the multivariable model, overweight or obese males featured a higher risk of IMV than lean men. This could not be replicated in females, suggesting that in DM, males could be more vulnerable to severe COVID-19 infection based on the underlying degree of fat mass. Whereas glycemic control was not related to death rates in either sex in this study, metformin therapy was associated with lower mortality risk only in men. In contrast, insulin therapy was associated with higher mortality in women. Furthermore, in an age- and BMI-adjusted linear analysis, plasma glucose at admission related to death in men only. Accordingly, following multiple adjustments, glucose levels above 180 mg/dL predicted death in men.

The fact that two-thirds of all hospitalized DM in the CORONADO study were males, mirrors the previously described male predominance of more severe disease regardless of the diabetes status. This is in line with reports from many different regions including meta-analyzes providing evidence of excess male death rates despite the comparable infection rates between the sexes. Similar effects were already observed during previous coronavirus outbreaks. In the latest report of the global sex-disaggregated data tracker it is very clear that despite more testing and vaccinations in women, the confirmed cases are sex-balanced (except those in healthcare-workers with 72% predominance in women), but the rates of ICU admission (64%) and death (57%) were much higher in men (globalhealth5050.org). Another analysis across 38 countries even found a 70% higher case fatality rate in males (8).

There are many possible explanations for sex disparities in pathophysiology and outcomes of COVID-19 but the exact underlying mechanisms and their interplay in various subgroups of patients including presence of different comorbidities like DM are still unclear (8, 9). Sex differences are mediated by differences in reproductive organs, sex hormones, genomic, and epigenetic effects which all impact the immune system and the innate and adaptive immune response to infections and vaccines (8). Estrogens, progesterone, and testosterone directly affect immune cells via signaling through their respective receptors. The combined effects of X-linked immune response genes and the sex steroid hormones estrogen and progesterone enhance immune tolerance and antibody production, and mitigate innate immune inflammatory response (8). Males consistently show greater proinflammatory cytokine production along with a more robust induction of non-classical monocytes whereas females develop more robust T cell activation during SARS-COV-2-infection (9). Women have higher levels of natural killer (NK) and B-cell as well as CD8+ T-cell numbers and activities. Poor T-cell response was associated with worse outcomes in men but higher levels of innate immune cytokines was related to more severe disease in women (9). Sex also affects the distribution of lymphocyte subsets and lymphocytopenia that may lead to worse COVID-19 disease progression. ACE2, which serves as receptor for cell entry for SARS-COV-2, is located on the X chromosome and downregulated by estrogens. Otherwise, TMPRSS2, a direct androgen receptor-target gene, is necessary for spike protein priming of the virus. Thus, both sex-biased ACE2 expression and regulation of TMPRSS2 by androgens may be involved in a different susceptibility to COVID-19, virus entry, and pathogenicity in men and women.

Contrastingly, gender-related sociocultural factors, household and childcare responsibilities potentially lead to pandemic-related stress and increased exposition in women. Differences in lifestyle, smoking habits, and pre-existing comorbidities also unequally affect the exposure and outcomes. Furthermore, men usually are prone to greater individual risk taking and less adherence to public health measures together with poor health-seeking behavior. Moreover, access to testing resources and healthcare may vary between sexes and is potentially limited for women in countries with high gender inequality. Type of occupation has an influence on exposure risks as well as women are more likely to work in the health-care sector. Therefore, a complex interplay of biological and behavioral factors, which may put men at greater risk, and gender-related psychosocial factors, which may disadvantage women, has to be considered. This interplay might explain why sex-disaggregated data concerning high-risk conditions such as obesity and DM are conflicting, ranging from higher risk in males to comparable risk between sexes, up to an increased risk in women, especially in DM2.

Limitations of the studies published until now include that all studies were observational* post hoc* analyzes and unequal groups of people/less women were included. No causal relationship can be deducted from these studies. In addition, some population-based studies included both hospitalized patients and outpatients with COVID-19 infection, thus, covering a wide range from mild to severe disease forms while others focused on patients admitted to hospitals or ICUs. Furthermore, information on comorbid autoimmune diseases, which are quite common especially in women with diabetes, could be clinically relevant but have not been reported in the published studies.

Taken together, based on observations from France, UK, US, and Austria, it could be hypothesized that women with diabetes lose the female biological advantage in terms of COVID-19 mortality compared to women without DM. In general, healthy women are more insulin sensitive and have a more favourable fat distribution and cardiometabolic risk profile with less low-grade inflammation. Protected by their gonadal hormones, women are less prone to develop DM at a younger age. However, DM prevalence is comparable between sexes at an older age (10). Lifelong interactions between biological sex and gender constructs differently impact the pathophysiology of DM in men and women. Women usually bear a greater burden of risk factors for DM, and may have a longer progression time until they develop diabetes, thus, generating more atherogenic load, like abdominal obesity/insulin resistance, inflammation, endothelial dysfunction, hypercoagulability, dyslipidaemia, or hypertension at the time of diagnosis (10). There is evidence that diabetes increases the relative risk of diabetic complications, especially vasculopathy, more dramatically in women than men. This leads to comparable and sometimes even greater cardiovascular risk in women among patients with DM, particularly at a younger age. Similar mechanisms might apply to COVID-19 infections. Unfortunately, no study has yet reported on menopausal status or possible effects of oral contraceptives or hormone replacement therapies on the course of COVID-19 in women with diabetes. Thus, more studies are needed to clarify whether and which sex differences exist in predictors of mortality in COVID-19 amongst patients with DM.

In addition, the use of different types of antidiabetic medication such as SGLT2 inhibitors or GLP-1 analoga, lipid lowering, antihypertensive, and anticoagulant treatments should be investigated regarding the potential sex differences in COVID-19 mortality or adverse events. In CORONADO, metformin was related to lower mortality in men. Indeed, the cytokine-reducing, anti-inflammatory, and sex-specific immunomodulating effects of metformin were suggested to be beneficial in another recent analysis, but only showed reduced mortality in women with obesity or DM2 in an outpatient setting (11). Up to now, no clear advantage of any specific antihyperglycemic class of drugs in patients with COVID-19 has been demonstrated, possibly due to differences in dose and time exposure as well as indication for prescription. Overall, the association between glycemic control, indicated by HbA1c, and mortality risk is inconsistent, while glycemia at admission might be of greater relevance for clinical outcome, including patients without previously diagnosed diabetes (5).

Another important sex-specific topic is COVID-19 in pregnant women, who feature higher risks of maternal and neonatal ICU admission, IMV, and other adverse perinatal outcomes compared with non-pregnant women of reproductive age (12). Women with pre-existing DM displayed an even greater, more than doubled, risk of severe outcomes. Furthermore, pregnant women with severe COVID-19 infections showed an almost double as high risk of developing pregnancy-related hyperglycemia, gestational diabetes, in a pooled analysis (12). Diabetes was associated with more than a four-fold and gestational diabetes with more than a three-fold higher risk of ICU admission, while diabetes was also related to maternal death.

In the high-risk group of pregnant women with DM, protection via COVID-19 vaccinations appears indispensable. However, up to now safety and efficacy data in this high-risk subgroup are lacking. Greater knowledge on immune response and potential adverse effects to COVID-19 vaccinations in the especially vulnerable group of pregnant DM would be needed. Vaccinations could also enable protection of the offspring via transplacental transfer of protective antibodies and via breastmilk.

Furthermore, sex-stratified analyzes of COVID-19 therapies including corticosteroids, immunotherapies, or antiviral medication, are still missing but necessary to further explore differences in outcomes between men and women. It is worrying that even in COVID-19 with obvious differences in vulnerability and acute and long-term outcomes between men and women, clinical trials are not designed to study sex differences. However, this kind of knowledge is essential for the development of innovative personalized treatments and more equitable health outcomes.

Concerning vaccines, vaccine efficacy appears to be comparable between sexes but adverse reactions, both mild allergic symptoms and anaphylaxis, are much more common in females. Parameters such as long-term protection, side effects, and development of sex-sensitive optimal dosing should be analyzed in respect to sex differences, especially for high-risk populations like patients with DM.

Moreover, men appear to be at a higher risk of acute complications and death, however, women may suffer more from long-term sequelae, especially chronic fatigue and neurocognitive and – psychiatric complications. So far data on prevalence and outcomes of 'long-COVID' among DM are missing but may be relevant as patients with DM generally have a higher risk of developing dementia, depression, and other psychiatric disorders with known sex differences (10).

In summary, an intersectional approach to COVID-19 research programmes and management with focus on sex and gender differences especially in vulnerable groups like DM is urgently needed. While men are obviously at higher risk of severe and fatal COVID-19, data among patients with DM are ambiguous so far. It appears that – comparable to findings on cardiovascular complications – women with DM may lose their biological female advantage resulting in comparable death rates to those of men. Knowledge on biological factors leading to functionally different immune responses and of gender-sensitive sociocultural determinants of COVID-19 infection rates may help to optimize prevention and management in both sexes. Innovative insights in sex and gender differences could guide the development of personalized strategies of prevention and care.

## Declaration of interest

The author declares that there is no conflict of interest that could be perceived as prejudicing the impartiality of this commentary.

## Funding

This work was supported by the Medical-Scientific Fund of the Mayor of Vienna (grant number CoVid004).
